# Acute Myeloid Leukemia with Isolated Trisomy 19 Associated with Diffuse Myelofibrosis and Osteosclerosis

**DOI:** 10.3390/cancers7040903

**Published:** 2015-12-14

**Authors:** Adam Stelling, Brian A. Jonas, Hooman H. Rashidi, Mehrdad Abedi, Mingyi Chen

**Affiliations:** 1 Department of Pathology and Laboratory Medicine, University of California Davis Medical Center, PATH Bldg. 4400 V Street, Sacramento, CA 95817, USA; abstelling@ucdavis.edu (A.S.); hrashidi@UCDAVIS.EDU (H.H.R.); 2 Division of Hematology and Oncology, University of California Davis Medical Center, Sacramento, CA 95817, USA; bajonas@ucdavis.edu (B.A.J.); mabedi@UCDAVIS.EDU (M.A.)

**Keywords:** acute myeloid leukemia, trisomy 19, myelofibrosis, osteosclerosis

## Abstract

Primary myelofibrosis (PMF), per WHO criteria, is a clonal myeloproliferative neoplasm that usually presents with a proliferation of granulocytic and megakaryocytic lineages with an associated fibrous deposition and extramedullary hematopoiesis. The bone marrow histologic findings of this disorder are typically characterized by the presence of myeloid metaplasia with an associated reactive fibrosis, angiogenesis, and osteosclerosis. However, marked myelofibrosis is not solely confined to PMF and may also be associated with other conditions including but not limited to acute megakaryoblastic leukemias (FAB AML-M7). Here, we describe a rare case of a non-megakaryoblastic acute myeloid leukemia with marked myelofibrosis with osteosclerosis and an isolated trisomy 19. A 19-year-old male presented with severe bone pain of one week duration with a complete blood cell count and peripheral smear showing a mild anemia and occasional circulating blasts. A follow up computed tomography (CT) scan showed diffuse osteosclerosis with no evidence of hepatosplenomegaly or lymphadenopathy. Subsequently, the bone marrow biopsy showed markedly sclerotic bony trabeculae and a hypercellular marrow with marked fibrosis and intervening sheets of immature myeloid cells consistent with myeloblasts with monocytic differentiation. Importantly, these myeloblasts were negative for megakaryocytic markers (CD61 and vWF), erythroid markers (hemoglobin and E-cadherin), and lymphoid markers (CD3, CD19, and TdT). Metaphase cytogenetics showed an isolated triosomy 19 with no JAK2 V617F mutation. The patient was treated with induction chemotherapy followed by allogenic hematopoietic stem cell transplantation which subsequently resulted in a rapid resolution of bone marrow fibrosis, suggesting graft-anti-fibrosis effect. This is a rare case of a non-megakaryoblastic acute myeloid leukemia with myelofibrosis and osteosclerosis with trisomy 19 that may provide insights into the prognosis and therapeutic options of future cases.

## 1. Introduction

Many benign and malignant hematologic conditions may be associated with an increase in bone marrow stromal reticulin or other collagen-type fiber deposition that can ultimately lead to myelofibrosis [1]. Among the myeloid neoplasms, as defined by the World Health Organization (WHO) classification, those associated with an increase in bone marrow fibrosis, include certain myeloproliferative neoplasms (MPN; e.g., primary myelofibrosis (PMF) polycythemia vera or essential thrombocythemia with secondary myelofibrosis, and BCR-ABL1-positive chronic myelogenous leukemia (CML)), myelodysplastic/myeloproliferative neoplasms (MDS/MPN; e.g., chronic myelomonocytic leukemia or the WHO provisional entity refractory anemia with ring sideroblasts and thrombocytosis), myelodysplastic syndrome (MDS; e.g., refractory anemia with fibrosis), and acute leukemia (e.g., acute megakaryoblastic leukemia and acute pan-myelosis with myelofibrosis) [2].

Other non-myeloid conditions (neoplastic and reactive) that can be associated with secondary marrow fibrosis which could be divided into two categories: one is secondary to infiltrative neoplastic disorders and the other is secondary to nonmalignant reactive conditions. The neoplastic conditions include Hodgkin lymphoma and non-Hodgkin lymphoma/leukemia such as hairy cell leukemia or mast cells disease and metastatic malignancies. The reactive conditions include infectious or granulomatous changes such as HIV, EBV, tuberculosis, fungal and leishmaniasis infection; autoimmune diseases such as systemic lupus erythematosus and sarcoidosis; treatment with hematopoietic growth factors such as thrombopoietin, IL-3 and IL-11; chronic inflammatory conditions such as inflammatory bowel disease or gastric bypass; metabolic or systemic disease such as chronic renal failure or Gaucher disease; chronic toxic myelopathies such as benzene poisoning, chemotherapy drugs, radiation damage and bone marrow transplant *etc.* [1].

Bone marrow biopsy sections are typically examined for stromal reticular fibers (also known as the type III collagen reticulin) using silver impregnation techniques (such as the Gomori’s stain) and the presence of type I collagen (including bone) through a trichrome stain [3]. Many studies have demonstrated that myelofibrosis is associated with abnormalities of the number and/or function of megakaryocytes and platelets [1,4]. Increased cytokines or growth factors secreted from megakaryocytes and platelets appear to play a critical role in myelofibrosis. In particular, platelet-derived growth factor (PDGF) and transforming growth factor-beta (TGF-β) are the most potent stimulators of fibroblast growth and bone marrow fibrosis [1,5]. Marked osteosclerosis and myelofibrosis are often seen in cases of hematologic malignancy but are seen most commonly in myeloproliferative neoplasms and acute megakaryoblastic leukemia (FAB-M7) especially in pediatric populations [6]. It was reported that interleukin-11 (IL-11), which is secreted from blasts, acts as an osteoprotegerin (OPG) inducing factor in patients with acute megakaryocytic leukemia and may lead to osteosclerosis [7]. In addition, trisomy 19 is frequently present in both entities; however, a definitive association between osteosclerosis and trisomy 19 has not been described [8]. Isolated trisomy 19 as the sole cytogenetic finding in a neoplasm is extremely rare and not specific to myeloid neoplasms [9]. Although this abnormality has been predominantly described in myeloid neoplasms (e.g., acute myeloid leukemia (AML), MDS, and MDS/MPN), it has also been found in acute lymphoblastic leukemia (ALL), plasma cell myeloma, adenocarcinoma and even a rare case of an astrocytic neoplasm [1,10,11]. Here we present a case of diffuse osteosclerosis in non-megakaryoblastic AML with isolated trisomy 19. The combination of myelofibrosis and osteosclerosis along with isolated trisomy 19 has not been previously described in non-megakaryoblastic AML to our knowledge.

## 2. Case Presentation

**Figure 1:**
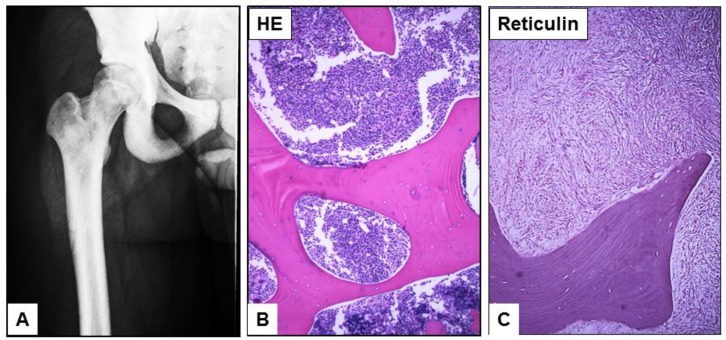
A bone scan shows diffusely hyperdense osseous structures in the femurs and pelvis bone. A computed tomography (CT) guided bone marrow biopsy showed markedly sclerotic bony trabeculae (**A**) with sheets of blasts (**B**) infiltrating the marrow space associated with myelofibrosis (**C**). ((**B**,**C**) magnification ×40).

A 19-year-old man presented with severe bone pain of one week duration with the CBC and peripheral smear showing mild anemia, thrombocytopenia and left shifted granulocytes with occasional circulating blasts (3%). A clinical concern for MDS was rendered in an outside hospital. The complete blood count (CBC) data shows WBC 7.9 K/μL RBC 4.00 M/ μL, HGB 12.2 g/dL, HCT 39.3%, MCV 98.3 fL, MCH 30.5 pg, MCHC 31.0 g/dL, and Platelets 76 K/uL. WBC differential: segmented neutrophils 49%, bands 4%, lymphocytes 28%, monocytes 2%, eosinophils 2%, basophils 0% metamyelocytes 3%, myelocytes 5%, promyelocytes 4% and blasts 3%. Notably, the lactate dehydrogenase (LDH), alkaline phosphatase (ALK), liver enzymes and the renal function studies were all within the normal limits. Multiple attempts at bone marrow aspiration were unsuccessful due to repeated dry taps. Computed tomography (CT) scans of the chest, abdomen, spine, and pelvis showed diffuse osteosclerosis with no evidence of hepatosplenomegaly or lymphadenopathy (Figure 1A). A CT guided bone marrow biopsy confirmed markedly sclerotic bony trabeculae within a cellular marrow (70%–80% cellularity) with sheets of immature myeloid cells/blasts (Figure 1B) associated with marked myelofibrosis (Figure 1C). 

**Figure 2:**
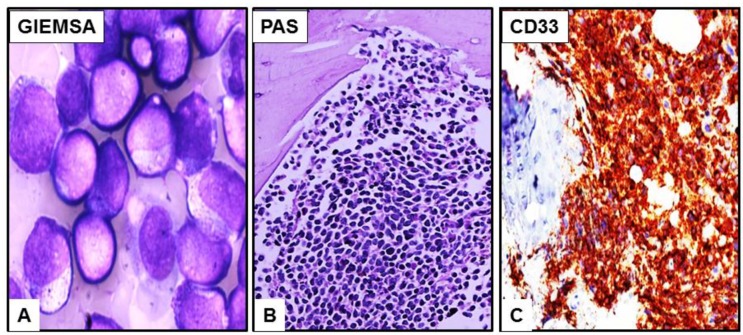
Bone marrow touch imprint reveals predominant blast population with increased N/C ratio, fine chromatin, prominent nucleoli and scanty cytoplasm with eosinophilic granules (**A**). In the core biopsy, the blasts are positive for PAS stain (**B**) and CD33 by immunohistochemistry (**C**). ((**A**), magnification ×600; (**B**)–(**C**), magnification ×100).

By flow cytometry, the hemodiluted specimen shows a small immature/blasts population with immunophenotype positive for CD33, CD13, dim CD4, dim CD64, CD11b, CD56, partial CD15, and myeloperoxidase (MPO). These same cells were negative for CD34, CD117, and HLA-DR. The blasts have round nuclei with high N/C ratio, fine chromatin, prominent nucleoli and scanty cytoplasm with fine eosinophilic granules (Figure 2A). No Auer rods were detected. No overt dysplasia was detected. In the core biopsy, the blasts were positive for PAS stain (Figure 2B). Immunohistochemistry of the blasts in the core biopsy demonstrated positive CD33 (Figure 2C), CD163, and MPO staining while megakaryocytic markers (CD61 and vWF), erythroid markers (hemoglobin and E-cadherin), and lymphoid markers (CD3, CD19, and TdT) were negative. 

Cytogenetics revealed a male karyotype showing a trisomy of chromosome 19 (47, XY, +19 (10)/46, XY (10)). There was no BCR/ABL fusion or RARA/PML t(15;17) translocation detected by FISH. By PCR, no JAK2 V617F mutation was detected; however, a FLT3-TKD mutation was detected. The overall presentation was consistent with an acute myeloid leukemia with monocytic differentiation. The negative expression of megakaryocytic markers made the differential diagnosis of acute megakaryoblastic leukemia (AML-M7) unlikely. The patient was treated with standard induction chemotherapy, consisting of 7 days of cytarabine and 3 days of daunorubicin (“7 + 3”), plus midostaurin, a FLT3 inhibitor, and achieved first complete remission (CR1). Allogeneic hematopoietic stem cell transplant (allo-HCT) was deferred in CR1. Unfortunately, the leukemia relapsed after 16 months of remission and the patient was retreated with 7 + 3 induction chemotherapy. After the second complete remission (CR2), he received an unrelated donor allo-HCT. Repeat bone marrow biopsy after the allo-HCT revealed a rapid resolution of bone marrow fibrosis, suggesting a graft anti-fibrosis effect, and restoration of marrow cellularity with trilineage hematopoiesis. The associated osteosclerosis was persistent and appeared irreversible. The patient has remained in CR2 over two years from the time of his allo-HCT and continues close clinical surveillance.

## 3. Results and Discussion

Bone abnormalities are commonly described in pediatric cases of acute leukemia and findings may include osteolysis, osteopenia, osteosclerosis, myelofibrosis, or mixed reactions [12]. These may mimic other orthopedic pathologies and careful workup with consideration for bone marrow biopsy is recommended [3]. In cases where osteosclerosis or myelofibrosis is prominent, it may be difficult to aspirate liquid marrow or obtain a trephine core biopsy of the bone marrow. In adults, osteosclerosis is not infrequently associated with hematologic malignancy and can be seen in certain myeloid and lymphoid neoplasms including, but not limited to, chronic myelogenous leukemia, primary myelofibrosis, acute megakaryoblastic leukemia, and hairy cell leukemia [13]. It may also be seen in some myelodysplastic syndromes in which confer a worse prognostic outcome [14]. A more detailed list of neoplastic entities with associated myelofibrosis is listed in Table 1. Although megakaryoblastic leukemias frequently show a gain of chromosome 19, the clinical characteristics and prognostic impact of non-megakaryoblastic AML with isolated trisomy 19 and diffuse osteosclerosis is likely a distinct entity that requires further investigation to elucidate its therapeutic and prognostic significance.

**Table 1:** Summary of hematolymphoid malignancies associated with marrow fibrosis.

Disease Name	Diagnostic Clinical Pathological Features
Primary Myelofibrosis	Intrasinusoidal clustered atypical/dysplastic megakaryocytic hyperplasia with myelofibrosis and osteoscelerosis
Acute Megakaryoblastic Leukemia (AML-M7)	Sheets of Blasts, acute onset, CD41+/CD61+
Acute myeloid leukemia, non-AML-M7 with fibrosis	Increased of myeloblasts >20%, usually CD34+/CD117+/CD33+
Acute Pan-myelosis with Myelofibrosis	Bone pain, acute onset without splenomegaly
Polycythemia Vera, Fibrotic Phase	Increased Hemoglobin, JAK2 mutation
Essential Thrombocythemia, Fibrotic Phase	History of thrombocytosis with mutations JAK2 (50%), MPL or CALR (5%)
Chromic myeloid leukemia, Fibrotic Phase	History of CML with BCR-ABL1+
MDS with Fibrosis	History of cytopenia and dysplastic features in marrow
Systemic mastocytosis	Atypical mast infiltrate with C-kit D816V mutation
Hairy cell leukemia	Diffuse Clonal B cells with B-raf mutation
Hodgkin Lymphoma with marrow fibrosis	Granulomatous changes with Hodgkin cells infiltrate (CD30+)
Follicular lymphoma	Paratrabecular nodular, clonal B-cell with t(14;18)/bcl2-IgH
Other B-cell lymphomas	Diffuse or interstitial, clonal B-cell infiltrate
Osteosclerotic plasma cells myeloma	Plasma cells with immunoglobulin light chain restriction

Since the major differential diagnoses in our case included an acute megakaryoblastic leukemia, primary myelofibrosis, and acute panmyelosis with myelofibrosis, these three entities are further discussed below:

### 3.1. Acute Megakaryoblastic Leukemia 

Acute megakaryoblastic leukemia (previously known as FAB AML-M7) is a WHO-2008 recognized subtype of acute myeloid leukemia, not otherwise specified (AML-NOS) where no specific recurrent cytogenetic abnormality is noted [2,15]. The diagnosis is based on the presence of 20% or more blasts with at least 50% showing an immunophenotype consistent with a megakaryocytic lineage [15]. This entity also has a notable association with myelofibrosis and osteosclerosis. The biopsy generally reveals dysplastic megakaryocytes, poorly differentiated blasts with cytoplasmic blebbing, cytoplasmic zonation, and positivity for CD61, CD41, and often CD42 by flow cytometry or immunohistochemistry [15]. It is important to make the distinction between *de novo* acute megakaryoblastic leukemia and other acute leukemias with megakaryoblastic phenotypes or osteosclerotic presentations including AML arising from MDS, AML evolved from chronic CML, and Down-syndrome related AML which may also have similar histopathologic features [6]. In the current case, given the chromosomal findings along with the histologic characteristics of the marrow (fibrosis and osteosclerosis), the possibility of an acute megakaryoblastic leukemia was ruled out by immunophenotypic analysis showing no megakaryocytic differentiation.

### 3.2. Primary Myelofibrosis (PMF) 

PMF is a subtype of myeloproliferative neoplasm (MPN) in WHO-2008 classification. It is characterized by a clonal myeloproliferation associated with variable osteomyelofibrosis. The mutations most commonly associated with PMF reside in the JAK2, CALR, and MPL genes [16]. Approximately 90% of PMF have one of these mutations while 10% carry none of these mutations [16]. It is a clonal disorder arising from the neoplastic transformation of hematopoietic stem cells in a constant crosstalk with their stromal environment, suggesting a deregulation of medullar stem cell niches in which hematopoietic stem cells are engaged [17]. The concomitant presence of abnormal angiogenesis and myeloid proliferation resulted in osteosclerosis [18]. The JAK2 V617F and JAK2 exon 12 mutations activate signaling that is mediated by cytokine receptors including erythropoietin, thrombopoietin, most interleukins, and interferon [19]. Recent progress in molecular biology reveals the common mutations (W515K and MPL W515L) of the MPL gene which encodes for the constitutively activated of thrombopoietin receptor which results in the overproduction of dysplastic megakaryocytes [16]. The abnormal megakaryocytes stimulate stromal cells to produce and accumulate collagen fibers in the bone marrow niches [1]. PMF is classified as a myeloproliferative neoplasm characterized by panmyelosis, clustered hyperchromatic megakaryocyte proliferation, and development of bone marrow fibrosis [16]. In the past, the same disease had alternate/historical names such as agnogenic myeloid metaplasia, chronic idiopathic myelofibrosis, and myelofibrosis with myeloid metaplasia [16]. In most instances, this entity remains as a diagnosis of exclusion. Notably, our current case didn’t meet the WHO-2008 diagnostic criteria for PMF which requires three major and at least two minor criteria [16]:

(a) *Major*: (1) Atypical densely clustered megakaryocytic hyperplasia with either fibrosis (MF-2 or 3) (fibrotic phase) or hypercellular marrow with granulocytic hyperplasia (cellular phase); (2) Does not meet criteria for PV, CML, MDS or other myeloid neoplasms; (3) Presence of JAK2 V617F or other clonal marker (e.g., MPL W515K/L or CALR), or if no clonal marker, exclusion of secondary causes of fibrosis.
(b) *Minor*: Splenomegaly, Leukoerythroblastosis, Anemia, and increase serum LDH.

### 3.3. Acute Panmyelosis with Myelofibrosis (APMF) 

APMF is a new entity in WHO-2008 and classified under acute myeloid leukemia not otherwise [2,15]. APMF can morphologically overlap with AML with myelofibrosis, primary myelofibrosis with evolving AML, and MDS with fibrosis [6]. APMF usually presents with pancytopenia in the peripheral blood with absent or only rare circulating blasts without overt anisopoikilocytosis [20]. Splenomegaly is absent, and extramedullary hematopoiesis in the spleen is not prominent [21]. The clinical course of APMF is rapidly progressive and fatal often terminating with an overt acute leukemia, therefore, it is essential to make the accurate diagnosis and distinguish it from its mimickers, particularly acute megakaryoblastic leukemia (AML-M7) through a detailed clinical history and hematological work up [20,22]. APMF usually shows hyperplasia of all three lineages with an increase in dysplastic megakaryocytes and frequently shows abnormal karyotypes [6]. In the current case, the morphologic feature of involvement in a single myeloid lineage in combination with the clinical scenario argues against a APMF.

Overall, the pathophysiology of bone marrow fibrosis in these disorders is not fully understood. Recent studies suggest that bone marrow fibrosis is more likely to be a result of the underlying cellular abnormalities rather than the primary process [19,23]. This evidence suggests it is due to a combination of different cytokines and growth factors that ultimately play a role in the fibrotic aspect of these disorders [23]. Increased number and/or function of megakaryocytes and platelets are commonly associated with myelofibrosis and are the likely key players in this pathologic process [16]. Cytokines from megakaryocytes and platelets appear to be necessary for fibrosis to occur [1,7]. Further studies have revealed a significant role of TGF-β in stimulating fibroblast collagen synthesis and pathologically increasing deposition of bone marrow stromal fibers [5]. It is also possible that other cell types and a variety of other cytokines and growth factors are also involved [1,5,24].

## 4. Conclusions

Chromosome 19 abnormalities may be present in any of these entities and may also occur as a secondary abnormality with additional recurrent abnormalities such as t(9;22), trisomy 21, t(1;22), as well as numeric abnormalities and deletions in MDS [25]. On the other hand, an isolated trisomy 19 is a marker of myeloid disorders most commonly associated with AML-M7 both in cases that are *de novo* and secondary to evolved MDS [8]. The frequent association of trisomy 19 with acute megakaryoblastic leukemia suggests genes encoded or regulated by chromosome 19 play a role in megakaryoblastic proliferation [10]. Additionally, acute megakaryocytic leukemia with osteosclerosis has been shown to have increased levels of an osteosclerotic cytokine, osteoprotegerin (OPG) [7]. The induction of OPG was demonstrated to be due to interleukin-11 production by the leukemic blasts [7]. The gene for interleukin-11 is located on chromosome 19 which may explain this association. Allo-HCT may exert a graft anti-fibrosis effect and significantly improve bone marrow fibrosis. A better understating of this intricate association may aid in future patient therapeutic and prognostic outcomes.
